# TMPRSS2-ERG Expression Predicts Prostate Cancer Survival and Associates with Stromal Biomarkers

**DOI:** 10.1371/journal.pone.0086824

**Published:** 2014-02-05

**Authors:** Christina Hägglöf, Peter Hammarsten, Kerstin Strömvall, Lars Egevad, Andreas Josefsson, Pär Stattin, Torvald Granfors, Anders Bergh

**Affiliations:** 1 Department of Medical Biosciences, Umeå University, Umeå, Sweden; 2 Department of Oncology-Pathology, Karolinska Institutet, Stockholm, Sweden; 3 Department of Urology, University of Gothenburg, Gothenburg, Sweden; 4 Departments of Surgery and Perioperative Sciences, Umeå University, Umeå, Sweden; 5 Department of Urology, Västerås Central Hospital, Västerås, Sweden; Innsbruck Medical University, Austria

## Abstract

The TMPRSS2-ERG gene fusion is found in approximately half of all prostate cancers. The functional and prognostic significance of TMPRSS2-ERG is, however, not fully understood. Based on a historical watchful waiting cohort, an association between TMPRSS2-ERG, evaluated as positive immune staining, and shorter survival of prostate cancer patients was identified. Expression of ERG was also associated with clinical markers such as advanced tumor stage, high Gleason score, presence of metastasis and prognostic tumor cell markers such as high Ki67, pEGFR and pAkt. Novel associations between TMPRSS2-ERG and alterations in the tumor stroma, for example, increased vascular density, hyaluronan and PDGFRβ and decreased Caveolin-1, all known to be associated with an aggressive disease, were found. The present study suggests that the TMPRSS2-ERG fusion gene is associated with a more aggressive prostate cancer phenotype, supported by changes in the tumor stroma.

## Introduction

Recurrent gene fusion between the androgen-regulated gene TMPRSS2 and members of the ETS transcription factor family, most commonly ERG, are present in about 50% of prostate cancer cases [1]. Presence of this fusion gene is a critical event in the development of prostate cancer [2]–[4]. Transgenic expression of the fusion gene however only results in PIN lesions and additional genetic changes, such as loss of PTEN and activation of the PI3K pathway, are needed to induce cancer [5]–[8]. Experimental studies overexpressing or repressing the fusion gene suggest that it promotes tumor cell invasiveness and cell proliferation [1]. Numerous studies have evaluated the association of TMPRSS2-ERG and outcome of prostate cancer patients with varying results [1]. A recent large cohort- and meta- analysis however indicates that fusion gene status is not an important predictor of prostate cancer mortality or recurrence in patients treated with radical prostatectomy [9]. To our knowledge, only two studies have examined fusion-genes status in relation to the natural course of the disease in a watchful waiting cohort. In both studies presence of the TMPRSS2-ERG fusion was associated with an increased risk of prostate cancer death [10], [11].

Fusion gene status is generally determined by fluorescence in situ hybridization (FISH). Positive epithelial ERG immune staining, using a recently developed antibody, is however highly correlated (95.7% sensitivity and 96.5% specificity) to the presence of TMPRSS2-ERG fusion gene [12]–[15] suggesting that immune staining could be a practical way to determine the presence of the TMPRSS2-ERG fusion gene.

Recent studies suggest that tumor aggressiveness is related to changes in the tumor microenvironment [16]. In prostate tumors the stroma is changed in relation to tumor aggressiveness [17]. During prostate tumor progression, cancer epithelial cells sends signals to the surrounding stroma, which thereby adapt to the needs of the growing tumor. The tumor stroma cells in turn sends growth promoting signals to the epithelium [17]. When different prostate cancer cells are incubated together with normal fibroblasts they induce changes among the fibroblasts that are tumor cell-line specific [18]. If this occurs also *in vivo* it could suggest that fusion gene positive and negative tumors may show differences in the tumor stroma. This has however to our knowledge never been examined. If this was the case it could help us identifying the largely unknown signals that determines the development of a tumor stroma associated with aggressive disease.

In this study we therefore examined a large historical cohort of TURP-diagnosed prostate cancers managed by watchful waiting by ERG immunostaining in order to explore whether ERG staining was associated with other tumor characteristics and long-term outcome, and in particular if it is associated with differences in tumor stroma morphology.

## Results

### Heterogeneous expression of TMPRSS2-ERG in tissue sampled from different tumor foci

To clarify the role of TMPRSS2-ERG in prostate cancer, a TMA containing material from 350 prostate cancer patients whereof 256 were managed with watchful waiting, was analyzed with ERG immunohistochemistry (IHC). The TMA contained 5–8 samples of tumor tissue and 4 samples of non-malignant tissue from different locations in the prostate of the same patient. Nuclear tumor ERG staining was observed in 34% of the patients. As previously shown, ERG expression sometimes varied when comparing different tumor foci from the same patient. Heterogeneous ERG staining was observed in 18% of the patients. In a few patients (6%) cytoplasmic ERG expression was found in epithelial non-malignant tissue. In all patients endothelial cells stained positive for ERG and this served as an internal positive control.

### TMPRSS2-ERG is associated with prognostic markers

The relation of TMPRSS2-ERG expression to already established histological and clinical prognostic markers was analyzed. Expression of ERG in at least one tumor core was significantly positively correlated with advanced tumor stage, high Gleason score and presence of metastasis. In addition, ERG expression was also associated with tumor epithelial cell markers such as high cell proliferation (Ki67) [19], pAKT [20] and pEGFR expression [21], all known to be related to poor outcome (Table 1 and 2). These experiments suggest that TMPRSS2-ERG is related to factors known to indicate poor prognosis for of prostate cancer patients.

**Table 1:** Bivariate correlations.

Tumor cell ERG IR					
	ERG positive (n)	ERG negative (n)	r	p	n
**Tumor stage**	135	208	0.299	<0.001	343
**Gleason score**	139	211	0.309	<0.001	350
**Presence of bone metastasis**	115	165	0.209	<0.001	280
**Ki 67 expression (epithelial)**	138	207	0.258	<0.001	345
**pEGFR (epithelial)**	105	159	0.195	<0.001	264
**pAKT (epithelial)**	112	157	0.217	<0.001	269

**Table 2:** Distribution of ERG positive patients related to prognostic factors.

	Fraction (%) of ERG positive patients
**Ki67 low**	**33**
**Ki67 high**	**67**
**Gleason 6**	**23**
**Gleason 8–10**	**51**
**pAkt low**	**29**
**pAkt high**	**71**
**pEGFR low**	**32**
**pEGFR high**	**68**
**Bone metastasis**	**24**
**T 1–2**	**61**
**T 3–4**	**39**

### TMPRSS2-ERG correlates with cancer specific survival

**Figure 1:**
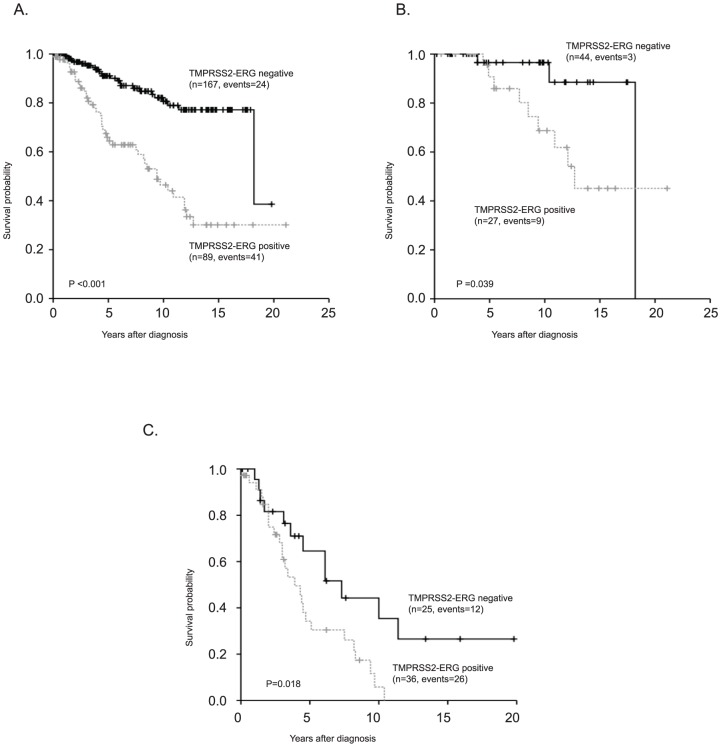
ERG expression in tumor cells predicts survival of prostate cancer patients. Patients are separated into two groups depending on presence of ERG expression (dashed line) or absence of ERG expression (solid line) in all patients (A), patients with Gleason score 6 (B), and patients with Gleason score 8–10 (C).

To evaluate the clinical significance of TMPRSS2-ERG in this cohort of patients followed with watchful waiting, survival analysis with Kaplan-Meier was performed. The analysis showed that patients with tumors expressing ERG had a significantly reduced survival as compared to patients with tumors lacking ERG staining (Figure 1 A). In addition, tumors expressing ERG in patients with Gleason score 6 or 7 tumors had significantly shorter cancer specific survival than those with tumors lacking ERG expression (Figure 1 B). A difference in survival between groups with tumors expressing ERG was also seen in patients with Gleason score 8–10 tumors (Figure 1 C). Tumors expressing ERG was associated with an increased relative risk for prostate cancer specific death in a univariate Cox regression analysis (Table 3). In multivariate Cox regression analysis including the established prognostic marker Gleason score and local tumor stage, presence of TMPRSS2-ERG in the tumor was significantly associated with poor prognosis and gave additional prognostic information (Table 4).

**Table 3:** Univariate Cox regression of tumor cell ERG IR of patients followed with watchful waiting.

Variable		n	RR	p-value	95% CI
**Gleason score** **	4–5	91	1*		
	6–7#	150	25.0	0.002	3.4–182.9
	8–10	63	128.7	<0.001	17.6–939.5
**Local tumor stage** **	T1a–T1b	189	1*		
	T2	74	4.0	<0.001	2.3–7.0
	T3	35	9.8	<0.001	5.4–17.8
	T4	3	11.6	0.017	1.5–88.1
**Tumor TMPRSS2-ERG** **	negative	167	1*		
	positive	89	3.8	<0.001	2.3–6.3

* Reference value.

** Cox regression analysis using Gleason score, local tumor stage and tumor ERG-IR as categorical variables.

Abbreviations: RR, relative risk; CI, confidence interval; immunoreactivity, IR.

# The group includes both Gleason grade 6 and 7 since the number of patients with grade 7 are too few to allow a separate analysis of this group.

**Table 4:** Multivariate Cox regression of tumor cell ERG IR of patients followed with watchful waiting.

Variable		n	RR	p-value	95% CI
**Gleason score** **	4–5	79	1*		
	6–7#	115	15.8	0.007	2.1–118.1
	8–10	59	61.0	<0.001	7.9–470.7
**Local tumor stage** **	T1a–T1b	152	1*		
	T2	68	1.5	0.229	0.8–2.9
	T3	30	1.7	0.201	0.8–3.9
	T4	3	2.9	0.320	0.4–22.6
**Tumor TMPRSS2-ERG** **	negative	166	1*		
	positive	87	1.9	0.019	1.1–3.3

* Reference value.

** Cox regression analysis using Gleason score, local tumor stage and tumor ERG-IR as categorical variables.

Abbreviations: RR, relative risk; CI, confidence interval; immunoreactivity, IR.

# The group includes both Gleason grade 6 and 7 since the number of patients with grade 7 are too few to allow a separate analysis of this group.

When patients were analyzed in 3 groups, ERG−, ERG+ and ERG heterogeneous (h) in Kaplan- Meier plot, ERG− had the most favorable prognosis, better than ERGh and ERG+, which had similar prognosis (data not shown).

### TMPRSS2-ERG is associated with stromal changes

The TMA have previously been employed to identify a number of prognostic markers. Stromal factors in prostate cancer that we previously identified as associated with survival of prostate cancer patients in this TMA are PDGFRβ [22], hyaluronan [23], Caveolin-1 (Scherdin et al, unpublished) androgen receptor [24], mast cells [25] and von Willebrand factor [19]. Interestingly, TMPRSS2-ERG was found to be associated with all of these factors, except AR and mast cells. In these studies we report that high stromal expression of PDGFRβ and hyaluronan, both in the tumor stroma and in the stroma of the surrounding non-malignant tissue, was associated with a poor outcome of prostate cancer patients. Additionally, high vessel density (measured as expression of von Willebrand factor) and decreased tumor stromal expression of Caveolin-1 was related to bad prognosis. TMPRSS2-ERG was found to associate with these factors in a manner predicting a poor outcome of the patient (high tumor stromal expression of PDGFRβ, hyaluronan, von Willebrand factor and low stromal expression of Caveolin-1) (Table 5 and 6). These results indicate that presence of TMPRSS2-ERG is related to stromal phenotypes associated with bad prognosis of prostate cancer patients.

**Table 5:** Bivariate correlations.

Tumor cell ERG IR			
Tumor stroma expression of:	r	p	n
**Hyaluronan**	0.208	<0.001	346
**PDGFRβ**	0.198	<0.001	261
**Caveolin-1**	−0.224	<0.001	346
**Von Willebrand factor (vascular density)**	0.249	<0.001	341

**Table 6:** Fraction of ERG positive patients related to expression of prognostic stromal factors.

	Fraction (%) of ERG positive patients
**Hyaluronan low**	**36**
**Hyaluronan high**	**64**
**PDGFRβ low**	**63**
**PDGFRβ high**	**37**
**Caveolin-1 low**	**71**
**Caveolin-1 high**	**29**
**Von Willebrand factor low**	**32**
**Von Willebrand factor high**	**68**

## Discussion

In the present study, TMPRSS2-ERG was found to be associated with a number of clinical parameters, including survival, in a patient cohort managed with watchful waiting. In addition to this, associations between fusion gene status and stromal genes that were previously identified as biomarkers with prognostic information in prostate cancer were also identified.

Current biomarkers to diagnose prostate cancer and predict prostate cancer outcome do not have sufficient specificity and sensitivity and generates problems with overtreatment and overdetection [26]. New and better prognostic markers to sort out patients in need of prostate cancer treatment is urgently warranted. The usefulness of TMPRSS2-ERG as a prognostic marker for prostate cancer has been heavily studied with different results. It is however becoming increasingly clear that in patients treated with radical prostatectomy, TMPRSS2-ERG fusion does not have a large impact on patient outcome [9]. Notably all studies, examining the outcome after watchful waiting (the natural cause of the disease) find that TMPRSS2-ERG is associated with a poor outcome. Prostate cancer is generally multifocal and in about 30% of men with prostate cancer their prostates harbor both fusion gene positive and fusion gene negative tumors [27], [28]. In such cases it is generally the fusion gene positive focus that forms lymph node metastases and in this study it was associated with the presence of bone metastases at diagnosis [27]. Hypothetically, presence of the fusion gene gives a more aggressive cancer only when patients are left untreated, possibly since the tumor needs time to acquire additional genetic changes, such as loss of PTEN [7], to be able to form macroscopic metastases.

Tissue materials generated with TURP might contain an overrepresentation of transitional zone tumors and since prostate cancers originating from the transitional zone are known to be biologically different from peripheral zone tumors this could influence the effect of ERG overexpression [29], [30]. The lower frequency of TMPRSS2-ERG in the present study (34%) as compared to approximately 50% in radical prostatectomy cohorts can also be explained by the patient cohort (the fusion gene is less frequent in transitional zone tumors) and is in line with what others have seen in TURP materials [9].

The finding that expression of the fusion-gene TMPRSS2-ERG in epithelial cells is related to stromal changes is novel and interesting. The tumor microenvironment, which is shaped by bidirectional communication between cancer epithelial cells and their surrounding stroma, is involved in all stages of cancer progression, holds prognostic information and affect response to treatment [31]–[33]. Interestingly, and in line with the present findings in prostate cancer, different breast cancer subtypes, based on their expression of estrogen- progesterone- and Her2-receptors in the epithelium, gives rise to stromal cells with different gene expression patterns and variable ability to support cancer cell migration [34]. All of the stromal changes that were found to associate with epithelial ERG expression are related to more aggressive cancer. Low stromal Caveolin-1 correlates with reduced relapse-free survival in prostate cancer patients and Akt activation [35]. Similarly, increased levels of PDGFRβ and Hyaluronan in prostate tumor stroma and surrounding non-malignant stroma associates with poor outcome of prostate cancer patients and injection of hyaluronan in the prostate increase prostate cancer growth in an orthotopic rat model [22], [23]. Angiogenesis is critical for the progression of prostate cancer, and presence of the fusion gene was associated with increased vascular density. To further investigate if and in what way cells expressing the TMPRSS2-ERG fusion gene is able to alter the tumor microenvironment and make it more hospitable to the tumor cells, would add important information on how TMPRSS2-ERG contributes to prostate cancer biology. Notably there are also changes in the tumor stroma related to tumor aggressiveness (for example decreased androgen receptor expression and mast cell numbers) that are apparently unrelated to epithelial ERG status. Cells carrying the fusion gene are known to have a specific gene expression pattern [36] and it might be possible to find key factors altering the stroma in a tumor promoting way, an effect that apparently adds aggressiveness to tumors of all Gleason grades.

## Conclusions

In this cohort, TMPRSS2-ERG was found to associate with a number of clinical parameters, including survival, related to poor outcome. This confirms previous results showing that presence of the fusion gene gives a more aggressive disease in patients left untreated. Moreover, this study has identified associations between TMPRSS2-ERG and stromal changes, previously identified as biomarkers predicting a worse prognosis of prostate cancer patients. This might indicate that the more aggressive phenotype that arises with the presence of TMPRSS2-ERG at least in part is caused by changes in the tumor stroma.

## Materials and Methods

### Tissue microarray

Tissue samples were collected from patients that underwent transurethral resection of the prostate (TURP) at the hospital in Västerås, Sweden, between 1975 and 1991. Prostate cancer was detected by histological analysis. Median age at time of diagnosis was 74 years (range 51–95 years). Information concerning presence of benign prostate hyperplasia was not available. Tissue specimens were formalin-fixed, paraffin-embedded and regraded according to the Gleason system by a pathologist in line with ISUP recommendations [37]. The tissue samples were used to construct a tissue micro array (TMA) using a Beecher Instrument (Sun Prairie, WI, USA). The edges of tissue fragments were avoided to prevent effects from chemicals and surgical devices used during the TURP procedure. The TMA:s contained 5–8 samples of tumor tissue representing both the primary and secondary Gleason grade and 4 samples of non-malignant tissue from each patient. The patients had not received anti-cancer therapy before TURP. Radionuclide bone scan was achieved after diagnosis for detection of metastases. 350 patients were included in the study, of which 256 patients were followed with watchful waiting after TURP. At symptoms from metastases patients received palliative treatment with androgen ablation and in a few cases radiation therapy or estrogen therapy, according to therapy traditions in Sweden during that time. Moreover, 94 patients that were treated with palliative treatment immediately after diagnosis were included in the analysis. Treated patients were not included in the survival analysis. The median overall survival for the patient group followed with watchful waiting was 5.9 years. 80 of the tissue samples were graded as Gleason score 4–5, 71 patients as Gleason score 6, 44 patients had Gleason score 7, and 61 patients Gleason score 8–10. 1 patient (0.4%) with Gleason score 7, and 8 patients (3.1%) with Gleason score 8–10 had bone metastases at diagnosis. In August 2003, 26 patients (10.2%) were still alive, 65 patients (25.4%) had died from prostate cancer and 165 patients (64.5%) had died from other causes.

Factors of potential prognostic significance such as Gleason score, tumor volume, tumor stage, tumor cell Ki67, pAKT and pERG expression as well as stromal factors such as androgen receptor, mast cells, PDGFRβ, hyaluronan, Caveolin-1 and von Willebrand factor had already been analyzed in the material and could be used in the present study (for references see above).

### Ethics statement

The material was collected according to Swedish regulations at a time when informed consent was not required. The research ethical committee at Umeå university hospital (Regional Ethical Review Board in Umeå) approved of the study and waived the need for consent.

### Immunohistochemistry

Tissue sections were deparaffinized and rehydrated in xylene, 99% ethanol, 96% ethanol, 70% ethanol (3×5 min in very step), and then washed in distilled water. The antigen was retrieved in Tris/EDTA (pH 9) for 1 h in a pressure cooker. Sections were then left to cool for 5 min in a water bath before being washed in first distilled water and then TBS for 20 min. The rest of the immunohistochemical procedure was performed in an intelliPATH FLX instrument (Biocare Medical, Concord, CA, USA) according to the manufacturers instructions. ERG antibody (CM421A, Biocare Medical) was diluted in Renoir Red (1∶50). MACH 3 Mouse HRP-Polymer (Biocare Medical) was used for detection. In the correlation analysis the samples were scored as positive for TMPRSS2-ERG if staining was detected in at least one of the tumor cores.

### Statistics

Correlations between nominal variables and continuous variables were analyzed using the Kendall's tau b correlation test. Data used in the correlation analysis was collected at the time of prostate cancer diagnosis. Patients included in survival analyses with the Kaplan-Meier and Cox regression were followed with watchful waiting. The duration of event-free survival (EFS) is defined as the time from TURP until the date of prostate cancer death, death of other causes, or if no death occurred, until the date of last follow-up. Differences in outcome between groups were tested with the log-rank test. The prognostic relevance of TMPRSS2-ERG immunoreactivity was examined by Cox regression analysis alone and together with Gleason score and and local tumor stage. The level of statistical significance was defined as *P*<0.05 (two-sided). Statistical analysis was performed using the SPSS 21.0.0 software for Os X (SPSS Inc., Chicago, IL, USA).
